# Identification of risk factors for the onset of delirium associated with COVID-19 by mining nursing records

**DOI:** 10.1371/journal.pone.0296760

**Published:** 2024-01-19

**Authors:** Yusuke Miyazawa, Narimasa Katsuta, Tamaki Nara, Shuko Nojiri, Toshio Naito, Makoto Hiki, Masako Ichikawa, Yoshihide Takeshita, Tadafumi Kato, Manabu Okumura, Morikuni Tobita

**Affiliations:** 1 Department of Healthcare Innovation, Juntendo University Graduate School of Medicine, Tokyo, Japan; 2 Department of Psychiatry, Juntendo University Faculty of Medicine, Tokyo, Japan; 3 Medical Technology Innovation Center, Juntendo University, Tokyo, Japan; 4 Clinical Research and Trial Center, Juntendo University, Tokyo, Japan; 5 Department of General Medicine, Juntendo University Faculty of Medicine, Tokyo, Japan; 6 Department of Emergency and Disaster Medicine, Juntendo University Faculty of Medicine, Tokyo, Japan; 7 Department of Cardiovascular Biology and Medicine, Juntendo University Faculty of Medicine, Tokyo, Japan; 8 Department of Respiratory Medicine, Juntendo University Graduate School of Medicine, Tokyo, Japan; 9 Tokyo Institute of Technology, Tokyo, Japan; Hosei University: Hosei Daigaku, JAPAN

## Abstract

COVID-19 has a range of complications, from no symptoms to severe pneumonia. It can also affect multiple organs including the nervous system. COVID-19 affects the brain, leading to neurological symptoms such as delirium. Delirium, a sudden change in consciousness, can increase the risk of death and prolong the hospital stay. However, research on delirium prediction in patients with COVID-19 is insufficient. This study aimed to identify new risk factors that could predict the onset of delirium in patients with COVID-19 using machine learning (ML) applied to nursing records. This retrospective cohort study used natural language processing and ML to develop a model for classifying the nursing records of patients with delirium. We extracted the features of each word from the model and grouped similar words. To evaluate the usefulness of word groups in predicting the occurrence of delirium in patients with COVID-19, we analyzed the temporal changes in the frequency of occurrence of these word groups before and after the onset of delirium. Moreover, the sensitivity, specificity, and odds ratios were calculated. We identified (1) elimination-related behaviors and conditions and (2) abnormal patient behavior and conditions as risk factors for delirium. Group 1 had the highest sensitivity (0.603), whereas group 2 had the highest specificity and odds ratio (0.938 and 6.903, respectively). These results suggest that these parameters may be useful in predicting delirium in these patients. The risk factors for COVID-19-associated delirium identified in this study were more specific but less sensitive than the ICDSC (Intensive Care Delirium Screening Checklist) and CAM-ICU (Confusion Assessment Method for the Intensive Care Unit). However, they are superior to the ICDSC and CAM-ICU because they can predict delirium without medical staff and at no cost.

## Introduction

The novel coronavirus disease (COVID-19) was declared a pandemic in 2019 by the World Health Organization (WHO) [1]. In Japan, more than 30,000,000 cases of COVID-19 have been reported since the first confirmed case on January 16, 2020 [2, 3]. This disease causes interstitial pneumonia and severe acute respiratory infections with high infection rates. The typical clinical symptoms of COVID-19 include fever, cough, diarrhea, and fatigue. However, atypical presentations include extrapulmonary involvement, such as gastrointestinal symptoms, multi-organ failure, and nervous system involvement. Moreover, there has been growing awareness of the neuropsychiatric manifestations of COVID-19 [4]; coronavirus affects the brain, leading to neurological symptoms such as delirium.

Delirium (sometimes referred to as an “acute confusional state”) is a common clinical syndrome characterized by disturbed consciousness, cognitive function, or perception, with an acute onset and fluctuating course [5]. Assessment of delirium, the most frequent clinical manifestation of acute brain dysfunction, is particularly important in patients with COVID-19 [6]. A review summarizing the incidence of delirium in patients with COVID-19 in 2021 showed that 29–40% of older adults developed delirium [7]. A recent case series from Wuhan, China, reported that 36% of patients with COVID-19 admitted to the hospital experienced neurological conditions, including altered mental states and ischemic strokes [4]. A retrospective study in Saudi Arabia found that one-quarter of patients with COVID-19 developed “confusion,” and almost 9% experienced seizures [8, 9]. Furthermore, COVID-19-associated delirium has been correlated with unfavorable outcomes, including prolonged hospitalization and elevated mortality rates [10, 11]. Several investigations have reported that delirium is prevalent in patients with COVID-19 and is associated with poor clinical outcomes in Japan [12, 13]. Consequently, the management of patients to avoid the onset of delirium is critical, with an escalating need to prognosticate its manifestation.

Delirium prevention is an important issue in COVID-19 infection; however, prevention strategies are nonpharmacological and resource- and personnel-intensive [14]. Current methods for identifying hospitalized patients at an increased risk of delirium require nurse-administered questionnaires with moderate accuracy [15]. However, it is not always possible to have sufficient medical personnel to implement such methods. In particular, during the early stages of the COVID-19 pandemic, the physical and human costs of the infection prevention measures were significant. Therefore, a method that does not interfere with daily operations is needed to identify patients at risk of delirium [16].

Recent significant advances have been made in machine learning (ML) technology, which has been effective in predicting the onset of various diseases [17–20]. Previous studies have used ML to predict the onset of mental disorders, such as depression and delirium [21–23]. These studies on delirium include predictions using ML with structured data, such as age, sex, and weight [16, 24–26], as well as predictions using unstructured text data provided by medical professionals [27–30]. However, a systematic review of predictive models for delirium found that none have been adequately and systematically evaluated [31]. Hence, we aimed to identify previously unknown risk factors for delirium that could be useful in predicting its onset. To achieve this, we used natural language processing (NLP) and ML techniques to analyze nursing records because they contain detailed information regarding patients’ daily conditions.

## Materials and methods

### Study design

The study period was from January 2021 to May 2021. This was a single-center, retrospective, observational study. Patients admitted to Juntendo University Hospital during the study period with COVID-19 registered as their disease condition were included. When this study was conducted, Juntendo Hospital (Tokyo, Japan) was an acute care hospital with 1,051 beds. The analysis of medical records was conducted from August 2021 to March 2023.

### Data source

In this study, patients with COVID-19 infection were diagnosed using the “Guidelines for the Clinical Management of COVID-19” [32]. Diagnoses were primarily confirmed using polymerase chain reaction (PCR), point-of-care (POCT), and antigen testing. Patient data were extracted from information provided by the Juntendo University Information Center. Any information that could identify individual participants was discarded or anonymized prior to the analysis. The extracted information included patient ID, date of birth, sex, medical history, free-text data, and occupation of the individual who described the text data. Notably, nursing records were identified as those recorded by nurses. Patients who (1) did not receive a confirmed diagnosis of infection via a PCR test and (2) were hospitalized for less than 48 h were excluded. In total, 273 patients were included in the analysis.

**Fig 1:**
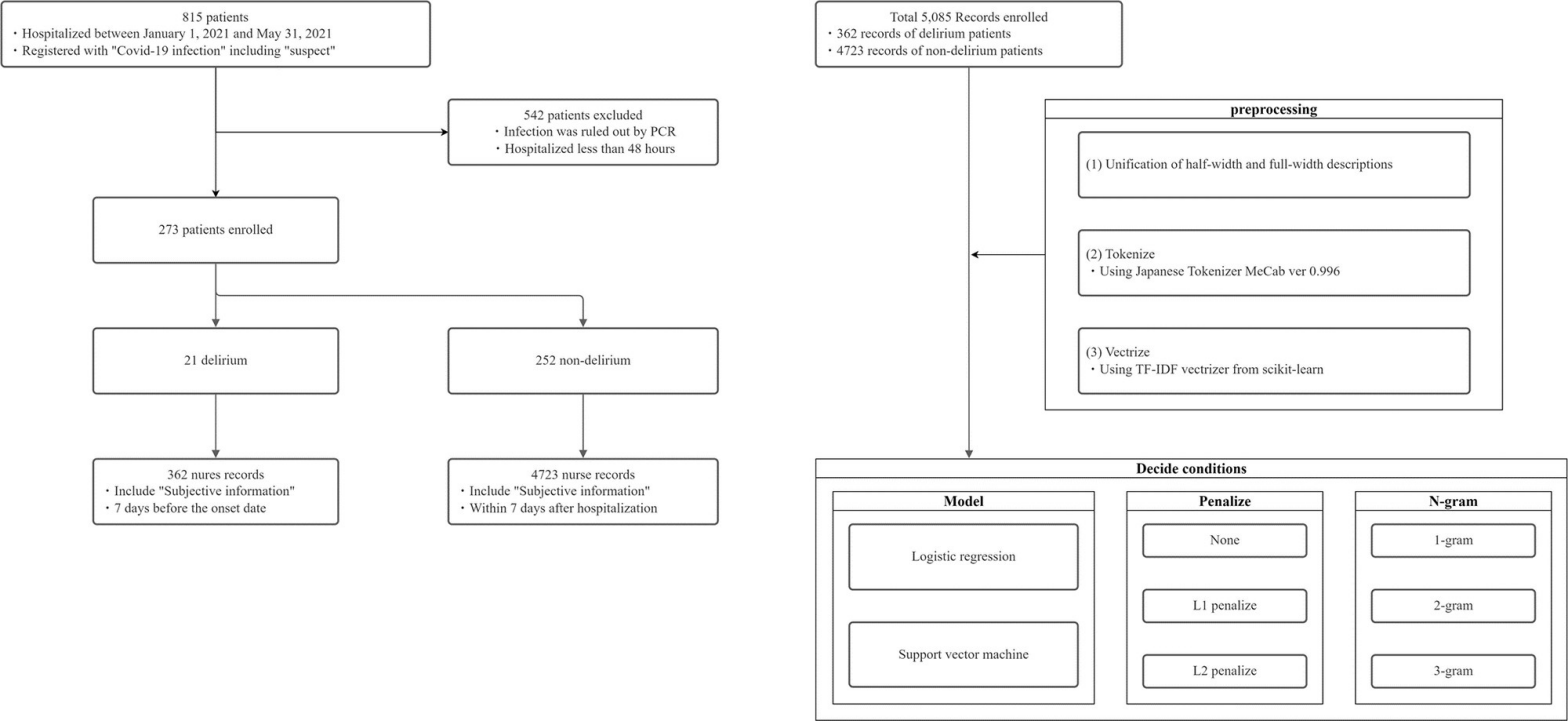
Flow diagram for the records included in this study.

Nursing records were written multiple times daily by different nurses. The records may be written in SOAP format or as free text. The SOAP format comprises subjective, objective, assessment, and planning information. Specifically, these included the patient’s condition, nurses’ observations, nurses’ review and assessment of vital signs, treatment, messages from the patient, and other comments from the nurse. Some nursing records only documented administrative information, such as prescriptions, and did not include the patient’s condition. This analysis aimed to uncover hidden risk factors through text mining, focusing on patients’ subjective information and nurses’ subjective comments. Therefore, nursing records that contained subjective information were included in the analysis. Fig 1 illustrates this process.

### Model development

Natural language texts cannot be used directly by ML algorithms. Therefore, they must be vectorized for processing using ML algorithms. The following are required to perform ML: (1) unification of half- and full-width characters, (2) breaking sentences down to the word level, and (3) vectorization. The text used in this study was written in Japanese. The Japanese language does not have word boundaries. Therefore, it was necessary to break down sentences at the word level. For this task, a Japanese morphological analyzer (MeCab [33]) was used to convert sentences into a standard form of segmented text. This text was then vectorized using the term frequency-inverse document frequency vectorizer from scikit-learn [34]. Through this process, text data written in Japanese were converted into a form that could be processed using ML algorithms. Fig 1 illustrates this process.

ML models were developed to analyze the nursing records of patients with delirium. Logistic regression (LR) [35], support vector machine (SVM) [36], decision tree (DT), and random forest (RF) were used as models. This was implemented using the scikit-learn package in Python [34]. The ML models employed in this study—LR, SVM, DT, and RF—have demonstrated high performance in identifying risk factors for delirium onset in previous studies [37–39]. The primary rationale for selecting these models is their interpretability in influencing the individual factors in the outcome. The objective of the present study was to identify key factors influencing delirium using the trained ML models; therefore, models that enable a clear interpretation of the relationships between factors and the disease are desirable. While some complex ML models, such as neural networks, are known for their high performance, they often pose challenges in interpreting the influence of individual factors. Consequently, the LR, SVM, DT, and RF models were chosen for their capability to quantitatively express the magnitude of the effect of each factor, making these models particularly useful for identifying risk factors for delirium. To optimize the performance of the ML model, we adjusted the parameters and evaluated the results. The model was then evaluated using the four-fold cross-validation method.

### Identifying risk factors

The features of each word were extracted from the trained ML model. A total of 50 1-gram words were extracted in the order of feature size. The Japanese language has the following ten parts-of-speech categories: verbs, adjectives, adjectival verbs, nouns, adverbs, participles, conjunctions, impressive verbs, auxiliary verbs, and particles. We defined function words, which include participles, conjunctions, impressive verbs, auxiliary verbs, and particles, as “non-significant words.” Non-significant words and words that appeared fewer than 100 times in the entire text were excluded. Moreover, 1-gram words with similar meanings were categorized into three groups. Subsequently, 2-gram and 3-gram words (in Japanese) were similarly classified into these three groups. This step was based on the opinions of the psychiatrists engaged in the clinical work.

### Evaluating risk factors using odds ratios

We identified the risk factors in the preceding steps. Next, we assessed the relationship between these risk factors and the onset of delirium by calculating odds ratios. We aimed to determine whether the words used by each of the three groups could predict delirium. The predictability of the words in each group was evaluated by determining the sensitivity, specificity, and odds ratio of words appearing (1) at least once a day or (2) at least twice a day regarding a particular patient.

### Measurement of study variables

The major outcome was delirium incidence. We tracked the presence or absence of delirium and date of onset. Delirium is often not registered in electronic medical records, even if there is an actual onset [40, 41]. Therefore, clinicians at the Juntendo Hospital Mental Clinic identified the onset date of delirium in patients. Experienced psychiatrists diagnosed delirium according to the DSM-5 criteria, based on patients’ medical history, physical examinations, and clinical test results, as well as physical and mental status examinations.

### Statistical analyses

Patient characteristics are described using the mean (SD) for continuous variables and percentages for dichotomous and categorical variables for all participants, patients with delirium, and patients without delirium. All analyses were performed using Python (version 3.10.5) and scikit-learn (version 1.1.3) [34].

### Ethics approval

As this was a retrospective study that dealt with existing medical records, the requirement for informed consent was waived. Information about the study design was posted on the Juntendo University Hospital website, and all candidates were guaranteed the opportunity to refuse to participate in the study. This study adhered to the “Ethical Guidelines for Medical Research for Humans” and the Declaration of Helsinki. The research protocol was approved by the Juntendo University School of Medicine Ethics Committee (approval no. H21-0102).

## Results

### Study population

The analysis included 273 patients, 21 (7.7%) of whom had delirium. The average age of all patients was 59.8 (SD, 19.1) years, and 61.1% were men. The average age of the patients with delirium was 73.5 (SD, 16.6) years, whereas that of those without delirium was 58.6 (SD, 18.9) years. Moreover, the length of hospital stay was 34.1 (SD, 27.7) and 12.6 (SD, 9.3) days for patients with and without delirium, respectively. The patient characteristics are presented in Table 1.

**Table 1:** Demographic characteristics of the patients and nurse records.

	Overall	Delirium	No delirium
N	273	21	252
Male sex	167	13	154
Age (decade, n)			
<10	2	0	2
10 s	7	0	7
20 s	17	1	16
30 s	18	0	18
40 s	32	0	32
50 s	44	5	39
60 s	51	1	50
70 s	59	5	54
80 s	39	6	33
90 s	4	3	1
Past medical history (%)			
Dementia	5	3 (14%)	2 (1%)
Epilepsy	14	1 (5%)	13 (5%)
Depression	10	1 (5%)	8 (3%)
Stroke	21	5 (24%)	16 (6%)
Insomnia	32	4 (20%)	28 (11%)
Hospital day, mean (SD)		34.1 (27.7)	12.6 (9.3)
Nurse record that includes subjective data to be analyzed, n	5085	362	4723

### Construction of the machine learning model

The model was developed using the nursing records of patients with delirium before the onset of delirium and those of patients without delirium as supervisory data. Specifically, the records of patients with delirium up to 7 days before admission were analyzed along with those of patients without delirium within 7 days after admission. The resulting nursing records totaled 5,085. Of these, 362 (7.1%) were nursing records of patients with delirium.

**Fig 2:**
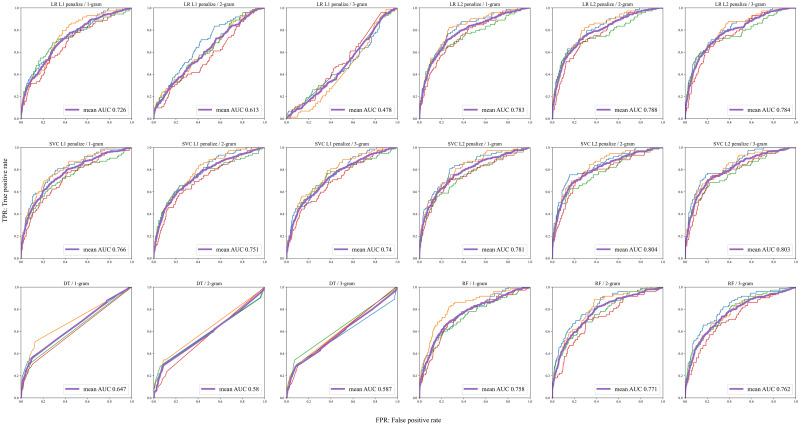
Graphs showing the construction of the machine learning model. LR, logistic regression model; SVC, support vector classifier; DT, decision tree; RF, random forest.

Next, ML models and the N-gram type (1–3-gram) were conditionally trained. The average area under the receiver operating characteristic curve (AUC) was obtained using a four-fold cross-validation method. Using LR, SVM, DT, and RF, the AUC was 0.478–0.788, 0.740–0.804, 0.580–0.647, and 0.758–0.771, respectively. In LR and SVM, the AUC tended to be smaller for L1 regularization. Moreover, no significant differences were observed in the AUC between the 1-gram, 2-gram, and 3-gram models. To identify many possible factor candidates, we decided to use a 3-gram model. According to a systematic review comparing predictive models for the development of delirium in older adults [31], the AUC of the predictive models ranged from 0.52–0.94. The results of the present study were within this range. The results are shown in Fig 2.

### Identifying risk factors

**Fig 3:**
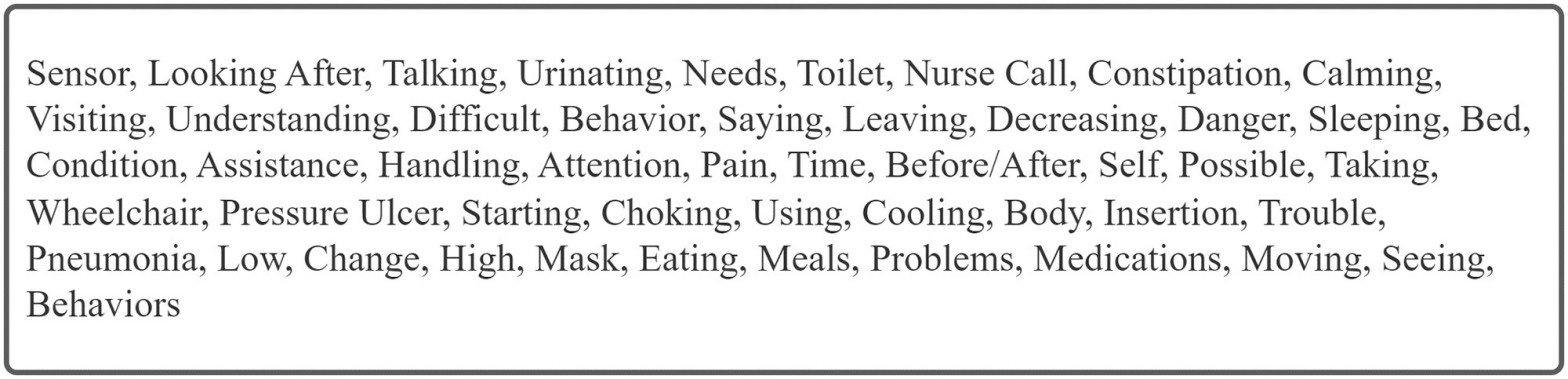
Fifty 1-gram words with high features were extracted from the machine-learning model.

**Fig 4:**
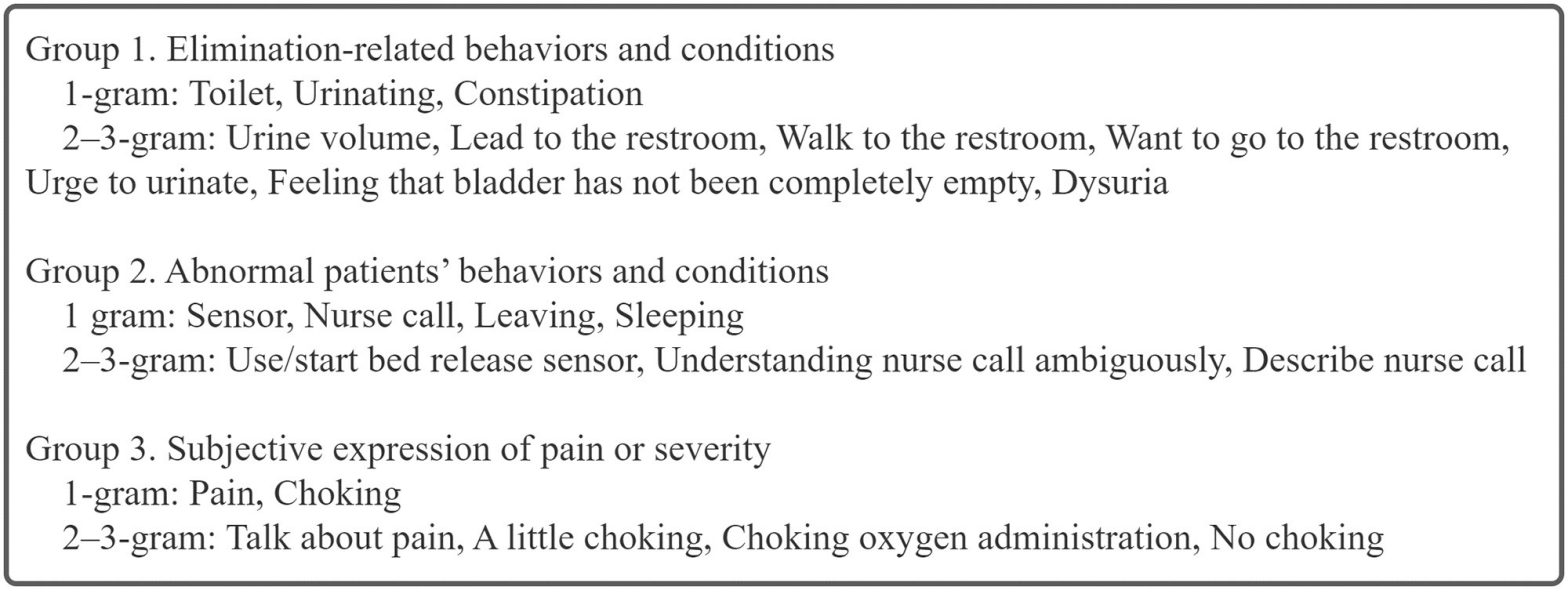
Grouping of risk factors (excluding non-significant words).

As described in the Methods section, we identified candidates for risk factors, and factors with similar meanings were consolidated into three distinct groups based on the opinions of the psychiatrists engaged in clinical work: (1) elimination-related behaviors and conditions, (2) abnormal patient behavior and conditions, and (3) subjective expression of pain or severity. A list of words is presented in Fig 3, and the word groups are shown in Fig 4.

### Prediction of disease onset using the risk factors

**Fig 5:**
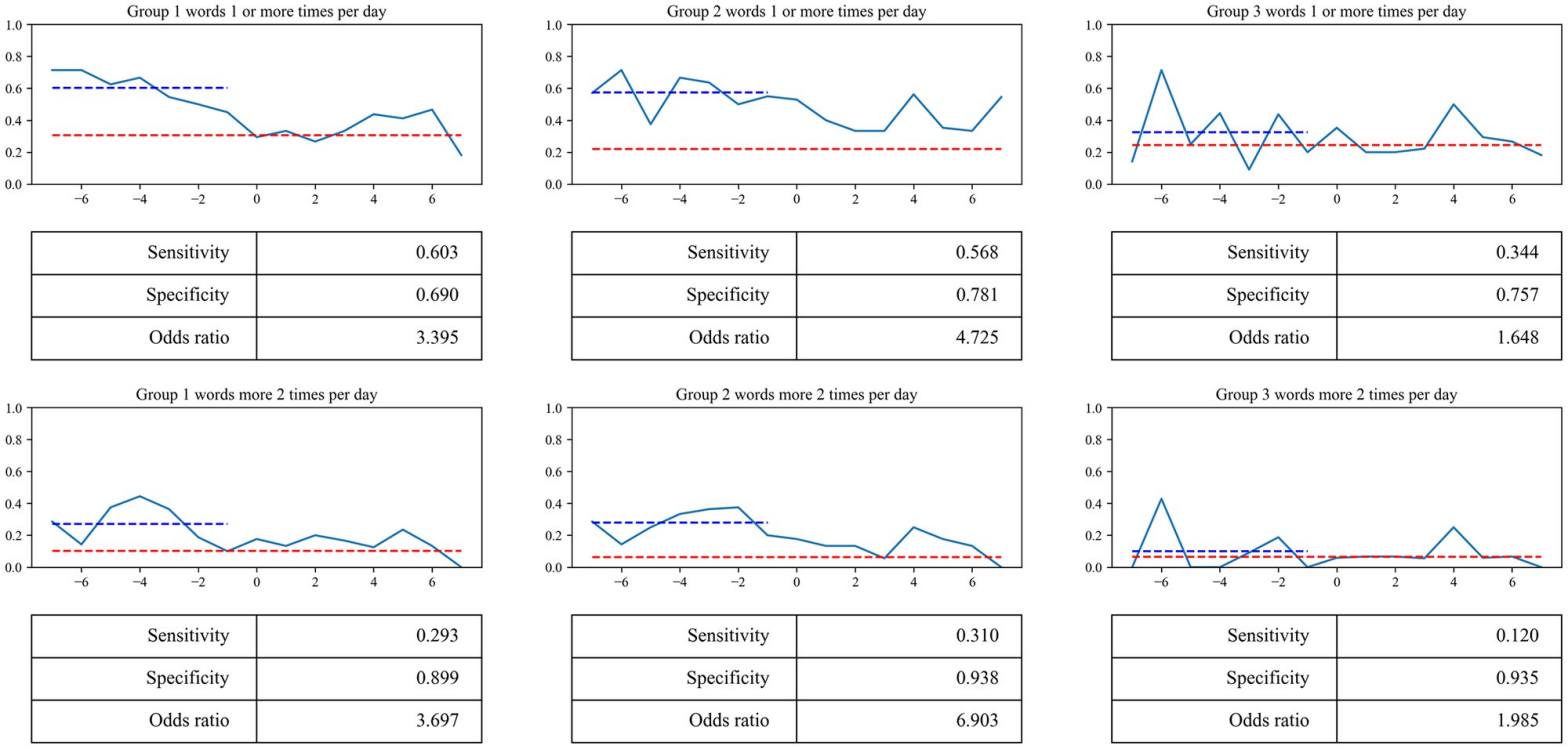
Graphs showing the sensitivity, specificity, and odds ratio for predicting disease onset using risk factors. The vertical axis represents the percentage of patients, and the horizontal axis represents 7 days before and after the day of delirium onset (day 0). The blue dotted line indicates the percentage of patients with delirium 1–7 days before its onset. The red dotted line indicates the frequency observed in patients without delirium.

We identified the risk factors in the preceding steps. Next, we evaluated whether these risk factors could predict delirium onset. For each patient, the number of times words in each group appeared per day was tabulated. For each group, we graphed (1) the frequency with which the word appeared at least once and (2) the frequency with which the word appeared at least twice. The sensitivity, specificity, and odds ratio for each group are shown in Fig 5.

Group 1 had the highest sensitivity (0.603). Group 2 had the highest specificity and odds ratio (0.938 and 6.903, respectively). However, group 3 had a low odds ratio (1.985). These results suggest that groups 1 and 2 may be useful in predicting delirium, but group 3 may not.

## Discussion

### Prior findings

Our objective was to identify unrecognized factors useful for predicting the onset of delirium using ML in the nursing records of patients with COVID-19. With this analysis, we identified (1) elimination-related behaviors and conditions and (2) abnormal patient behavior and conditions as predictors of delirium. In contrast, (3) subjective expression of pain or severity was not a predictor.

However, the identified factors were less sensitive than assessment methods often used in daily practice. Representative assessment methods for delirium include the Intensive Care Delirium Screening Checklist (ICDSC), Confusion Assessment Method for the Intensive Care Unit (CAM-ICU), Delirium Rating Scale-Revised-98 (DRS-R-98), and Memorial Delirium Assessment Scale (MDAS) [42, 43]. In particular, CAM-ICU and ICDSC are often used [44]; CAM-ICU has a sensitivity of 0.771–0.826 and specificity of 0.948–0.968, and ICDSC has a sensitivity of 0.653–0.815 and specificity of 0.767–0.864 [45]. Group 1 demonstrated the highest sensitivity (0.603) but low specificity (0.690). In contrast, group 2 demonstrated the highest specificity (0.938) but low sensitivity (0.310). Compared to using previous methods, prediction using the risk factors identified in this study may not be immediately effective.

However, these assessment methods require additional work for medical staff, and our predictors can be objectively and automatically collected without additional work. Under pandemic conditions, the ability to predict the onset of delirium without additional work and direct contact is desirable, as it can reduce the use of physical resources for infection prevention and risk [16]. In clinical practice, a more detailed evaluation, such as ICDSC, should be performed when these risk factors are identified in patients. This would effectively enable assessment of the risk of delirium, and necessary management to prevent onset can be performed in advance.

### Predictors identified in this study

Group 1, comprising elimination-related behaviors and conditions, included words such as “toilet,” “urination,” and “constipation.” Some studies suggest that elimination is associated with delirium. A lack of physiological control may exacerbate confusion or be considered a symptom. Among the risk assessments for delirium, the NEECHAM Confusion Scale includes an item on “urinary function” [46]. Furthermore, aspects of elimination, such as diaper use, urinary incontinence, and urinary retention, are useful for delirium risk assessment [42, 47, 48]. According to one of the authors, a psychiatric clinician, patients with mental instability occasionally complain of indefinite urinary symptoms. More detailed data collection should be performed in the next step, such as when and how many patients visit the toilet, as it may enable the early prediction of delirium onset.

Group 2, comprising abnormal patients’ behavior and conditions, included words such as “sensor” and “nurse call.” The use of bed sensors or bed fences is a risk factor for delirium development [47, 49], restricting a patient’s body movements [49]. Immobility is thought to be a risk factor, so it encourages medical staff to perform active range-of-motion exercises [5, 42, 48, 50]. To the best of our knowledge, no prior research has been conducted on the relationship between nurse calls and delirium. However, one study statistically summarized the frequency and content of nurse calls, with calls related to the “toilet” being the most common [51]. Given that elimination-related complaints were considered potential risk factors in this study, an increase in nurse calls may reflect these concerns.

Group 3, comprising subjective expression of pain or severity, included words such as “pain” and “choking.” Patients with severe COVID-19 have been reported to develop delirium [4]. Moreover, patient conditions, such as “shortness of breath,” “fever,” “insomnia,” and “catatonic state,” are related to delirium [42, 48, 52–55]. However, in the present study, the relationship between the symptoms and the onset of delirium was weak. One reason for the difference from previous studies may be that the expressions “pain” and “choking” alone do not capture the severity of the symptoms. Objective indices are appropriate for evaluating disease severity. For example, “shortness of breath” should be evaluated using SpO_2_ [5, 15]. However, whether scaling subjective symptoms is useful in predicting disease onset remains to be examined.

### Change in the frequency of occurrence of predictors before and after delirium onset

We assessed the relationship between these risk factors and the onset of delirium by calculating odds ratios. Fig 5 compares the frequency of occurrence of these factors before and after delirium onset. According to this graph, the frequency of occurrence of words in Groups 1 and 2 was higher before the onset of delirium than in patients without delirium. This demonstrates that the risk factors in Groups 1 and 2 were related to the onset of delirium. However, after the onset of delirium, the frequency decreased and approached that of patients without delirium. We hypothesized that one of the reasons for the decrease after onset may be that therapeutic interventions improved the severity of delirium. Another possible reason for the decrease after onset could be that the therapeutic intervention led to changes in patients’ complaints. For example, if a urinary catheter is inserted as a therapeutic intervention, the frequency of requests to go to the toilet is expected to decrease. Since our study could not examine this aspect, we cannot draw a definitive conclusion.

### Strengths and limitations

Our study had several strengths. First, we employed a text mining technique to analyze free-text nursing records. This approach allowed us to successfully identify hidden risk factors such as (1) changes in elimination-related behaviors and conditions and (2) increased frequency of nurse calls and sensor alerts, which were not represented in a structured format in electronic health records. Therefore, the strength of this study lies in its ability to discover findings that cannot be detected by analyzing structured data.

Furthermore, our results can ease the workload of medical staff. Traditional methods, such as ICDSC and CAM, require additional work for patient assessment. For instance, CAM, which is a representative assessment method, requires approximately 5 min [56]. This may overwhelm the staff’s daily work if the number of patients is high. In contrast, the risk factors identified in this study can be collected without additional work. For instance, by developing sensors that detect nurse calls or when a patient leaves bed, data can be collected without human intervention. If the collected data meet predefined criteria, alerts can be triggered, thereby identifying patients at high risk of developing delirium before its onset. Early identification facilitates timely intervention, potentially preventing the worsening of outcomes due to delirium. In terms of clinical applications, we envision a product concept of a compact sensor that can fulfill all the necessary functions and be easily attached to the bedside. Such devices can be readily introduced into clinical settings. Moreover, such a device would not increase the chances of contact with patients. During pandemics, factors such as a shortage of human resources make executing standard delirium management practices in routine clinical settings challenging [16, 57]. Another major challenge in the early stages of the COVID-19 pandemic was the risk of spreading infection through contact. We believe that this study offers a solution to this challenge.

However, our study had some limitations. The data used in this study were obtained from a single hospital over a short period. Therefore, our results may not be generalizable. In addition, we used nursing records written in Japanese. Therefore, similar results cannot be obtained from nursing records written in other languages. External validation is required to ensure the generalizability of our results.

### Future direction

In this study, we investigated the incidence of delirium. However, NLP and ML have been effective for diseases other than delirium [22, 58]. Additionally, text mining for psychiatric disorders is also effective in assessing depression [21, 58]. Our method may be used to identify predictors of the onset of other psychiatric diseases.

LR, SVM, DT, and RF were used as ML methods. This was done to emphasize the ease of interpreting the results. In general, neural networks and deep learning are effective in improving ML performance [59, 60]. Moreover, Bidirectional Encoder Representations from Transformers have demonstrated high performance in NLP tasks [18, 61]. Ensemble learning, which combines multiple ML methods, is also effective [62, 63]. Furthermore, by adding age, sex, severity of illness, and progress in daily laboratory findings in future research, better prediction performance can be expected.

The data used in this study were limited. An important step in the future would involve applying the predictive keywords and NLP algorithms identified in this study to data from many other hospitals over a long study period. Obtaining a larger dataset from multiple hospitals and sites over a longer study period may help overcome overfitting and bias.

## Conclusions

This study successfully employed NLP and ML techniques to analyze nursing records, leading to the identification of specific risk factors for the onset of delirium in hospitalized patients. Our analysis revealed two key predictors: (1) changes in elimination-related behaviors and conditions and (2) increased frequency of nurse calls and sensor alerts. These findings are significant as they can provide a novel, automated approach to delirium risk assessment without imposing additional workload on medical staff. The identified risk factors not only enhance our understanding of early delirium indicators but also offer a practical tool for early intervention, potentially improving patient outcomes in clinical settings.
